# Breaking bad in the rice field by breaking the Hox code

**DOI:** 10.1093/nsr/nwaa158

**Published:** 2020-07-20

**Authors:** Yoshinori Tomoyasu

**Affiliations:** Department of Biology, Miami University, USA

The beautiful green carpet of the early summer rice field is a sight that many people admire in Asian countries. But within the rice paddy is where planthoppers, one of the most serious insect pests in Asia, grow and outbreak [1]. Considering that rice is the staple food source in Asia, feeding up to 50% of the world's population [2], it is crucial to understand the biology of planthoppers to be able to better control these hideous pests. An intriguing aspect of their biology is the presence of two forms: the short-winged and long-winged forms. planthoppers switch between these two forms in response to environmental conditions [3]. When the rice plantharbors rich nutrition, the short-winged planthoppers dominate and consume the plants. Once the rice matures and the plant begins to dry out, this signals the planthoppers to become the long-winged form, allowing them to fly away to seek a better habitat. This environment-dependent two-form system (i.e. polyphenism) is critical for planthoppers to maximize their survival and reproduction. Some significant progress has been made in the past decade on the molecular mechanism underlying this polyphenism, including the two-insulin-receptor system [3,4] and glucose-triggered morph switching [5]. Now, in a recent issue of *National Science Review*, Liu *et al.* report an unexpected new player in the planthopper wing polyphenism: the Hox gene *Ultrabithorax* (*Ubx*) [6].

All winged insects are considered to have evolved from an ancestral insect that had two pairs of relatively unmodified wings (similar to modern-day dragonflies) [7]. These two pairs of wings have been evolutionarily modified uniquely in each lineage, allowing them to pursue various niches. For example, the hindwing of flies has been modified into the haltere, a dwarf balancing organ, while the forewing of beetles has evolved into the elytron, a protective shield. These modifications are determined by the segment-specific function of Hox genes, including *Ubx* [8,9]. It is a well-established paradigm that *Ubx* is the ‘hindwing’-selector gene across various insect groups, from flies, beetles and butterflies to milkweed bugs [8,9].

Liu *et al.* first performed RNA interference (RNAi) to knock down *Ubx* in planthoppers. The hindwing of planthoppers is smaller than the forewing in both short-winged and long-winged forms. *Ubx* RNAi induced hindwing-to-forewing transformation, resulting in the enlarged hindwing in both forms of planthoppers, demonstrating that the hindwing-selector function of *Ubx* is conserved in these insects. To their surprise, however, *Ubx* RNAi also induced enlargement of the forewing in the short-winged form of planthoppers. This outcome suggests that *Ubx* might have gained a new function in regulating the size of the forewing in the planthopper lineage. Expression analysis revealed that, although predominantly expressed in the hindwing, *Ubx* is also expressed in the forewing of planthoppers. Moreover, the *Ubx* expression is higher in the short-winged form than in the long-winged form, and higher in individuals infested on the growing rice plant than in those on the matured rice plant. The authors also performed an RNAi-based epistatic analysis, demonstrating that *Ubx* expression is regulated by two antagonistic insulin receptors (NlInR1 and NlInR2), downregulated by NlInR1 and upregulated by NlInR2. Together, these results reveal an intriguing evolutionary event in which *Ubx*, whose original function was to determine the hindwing identity, has been co-opted to produce the short-winged form via restricting the size of both forewings and hindwings in response to environmental cues (such as maturation of the host plant).

The impact of the findings reported by Liu *et al.* is two-fold. First, from a pest-control point of view, the identification of new factors that are involved in the wing-form regulation in planthoppers will provide more targets to control these pests. Second, from an evolutionary and developmental biology point of view, this study illustrates a fascinating case in which the expression of a Hox gene has not only been co-opted into a new context (i.e. forewing), but also become ‘plastic’ to facilitate polyphenism in response to environmental cues. Interestingly, at least in a laboratory condition, the two forms can be fixed into stable strains, suggesting that the environmental-cue-detecting system and the two-form-producing system are separable. A more in-depth analysis of how *Ubx* expression is regulated both environmentally and genetically in planthoppers will provide novel insights into the molecular basis underlying the evolution of polyphenism.

***Conflict of interest statement.*** None declared.
